# 3D planning and patient specific instrumentation for intraarticular corrective osteotomy of trapeziometacarpal-, metacarpal and finger joints

**DOI:** 10.1186/s12891-022-05946-x

**Published:** 2022-11-08

**Authors:** Method Kabelitz, Pascal Raffael Furrer, Sandro Hodel, Sandro Canonica, Andreas Schweizer

**Affiliations:** 1 Department of Orthopedics, Balgrist University Hospital, University of Zurich, 8008 Zurich, Switzerland; 2 Department of Hand Surgery, Balgrist University Hospital, University of Zurich, 8008 Zurich, Switzerland

**Keywords:** Correction, Intra-articular, Osteotomy, Patient-specific instrumentation, Finger osteotomy

## Abstract

**Background:**

Intra-articular malunions of the finger can lead to deformity and loss of function and can be treated with intra-articular corrective osteotomies. The aim of this study was to evaluate radiographic joint congruency, feasibility and functional outcome of three-dimensional (3D) printed patient-specific instrumentation (PSI) for corrective osteotomies at the trapeziometacarpal and finger joints.

**Methods:**

Computer-tomography (CT) scans were acquired preoperatively for standard 3D planning, which was followed by calculation of cutting planes and the design of individualized bone surface contact drilling, sawing and reposition guides. Follow-up CT scans and clinical examinations (range of motion, grip strength) were performed. Postoperative complications were documented and patient-reported outcome measurements were assessed (Single Assessment Numeric Evaluation (SANE) score, brief Michigan Hand Questionnaire (MHQ)).

**Results:**

Ten patients (mean age 28.4 ± 12.8,range 13.8–51.3) years) were included with a mean follow-up of 21 ± 18 (3–59) months including seven osteotomies at the trapeziometacarpal or metacarpophalangeal joints and three at the proximal interphalangeal joint (PIP). All radiographic follow-up examinations showed the planned correction with good joint congruency and regular osseous consolidation. At the latest follow-up, the range of motion (ROM) increased and the average grip strength recovered to the level of the contralateral side. No postoperative complication was detected. The mean SANE score improved from 44 ± 23 (0–70) to 82 ± 12 (60–90) after a mean of 72 ± 20 (44–114) months. The mean postoperative brief MHQ was 92 ± 8 (71–98).

**Conclusion:**

The use of 3D PSI in treating intra-articular malunions at the trapeziometacarpal and finger joints restored articular congruency accurately. ROM and grip strength improved postoperatively comparable to the healthy contralateral side and patient-reported outcome measures improved after medium-term follow-up.

## Background

Intra-articular fractures of the metacarpophalangeal (MC)- and finger joints can lead to serious disability, pain, reduced range of motion (ROM) and severe functional impairment if treated incorrectly [1, 2]. Posttraumatic or postoperative malposition of fragments with incongruency of the joint can result in posttraumatic arthritis [3]. In recent years, new technologies have been developed to address posttraumatic and postoperative malposition of intra-articular fractures [4, 5]. Corrective osteotomies with the aim of restoring normal anatomy are indicated when posttraumatic bone deformities become symptomatic [4, 6]. In combination with three-dimensional (3D) planning tools, the use of patient-specific instrumentation (PSI) in corrective osteotomies of intra-articular malunions can restore joint congruency with high precision [4].

While the benefit of PSI has been demonstrated for malunions at the wrist, and at the extra-articular phalanges [4, 7, 8], the role of PSI for treating intra-articular malunions at the finger joints remains unclear.

The aim of the study was to evaluate radiographic joint congruency, feasibility and functional outcome following 3D printed PSI for corrective osteotomies of intra-articular malunions in trapeziometacarpal (CMC I) and finger joints.

## Methods

### Study cohort

All patients who underwent intra-articular corrective osteotomy of an intra-articular malunion of the CMC I- or finger joint between February 2015 until August 2020 were included for this retrospective single center case series. Previous trauma to the contralateral hand would have been an exclusion criteria. All patients presented with a symptomatic painful malunion and impaired ROM and function. Radiographic indications, which at the same time represents an inclusion criteria, to correct the deformity comprised a minimum intra-articular step-off of 1.5 mm, a malrotation of more than 10° (phalangeal fracture) or a combined step-off and subluxation (CMC I fracture). In three patients malunion occurred after a previous surgery performed at other institutions (open or closed reduction with wire or screw fixation). All other included fractures were primarily treated non-operatively. Baseline characteristics, including handedness and previous surgical or nonsurgical interventions of the affected joint, were retrieved from patient charts. Written informed patient consent and approval of the local ethical committee (BASEC No. 2021–00052) were obtained. The study was conducted in accordance with the “Strengthening the Reporting of Observational Studies in Epidemiology” (STROBE) guidelines [9].

### 3D preoperative planning

**Fig. 1:**
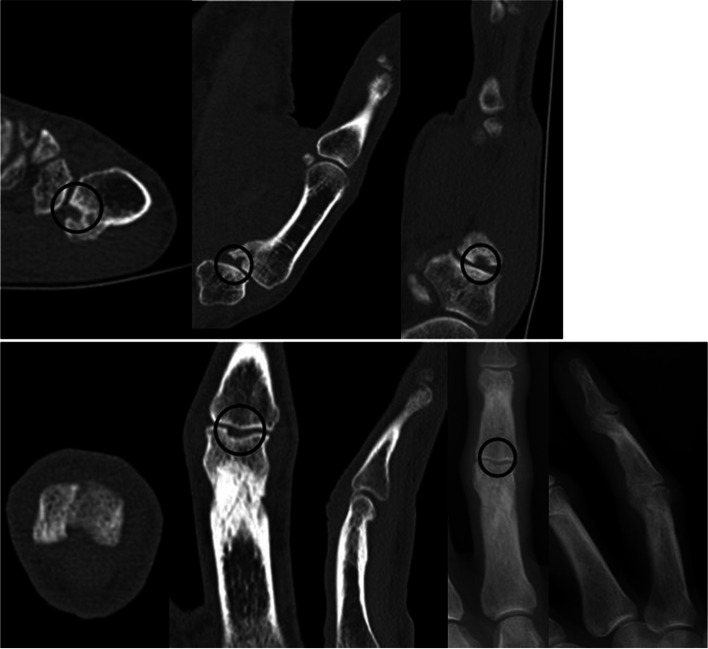
Top: Preoperative CT (left) and conventional radiographs (right) of malunited intra-articular fracture of the proximal interphalangeal III joint; bottom: Preoperative CT of malunited intra-articular fracture of the proximal metacarpophalangeal I joint

Negative trauma history of the unaffected side was reassured before acquiring the imaging required for pre-interventional planning. Preoperative computer-tomography (CT) scans and conventional radiographs were acquired of the affected and the contralateral side (Fig. 1). Radiological imaging was analysed using IMPAX (version 6.4.0.6010, Agfa-Gevaert N.V., Mortsel, Belgium). Software-based segmentation was performed using Mimics (Materialise, Gilching/Munich, Germany).

**Fig. 2:**
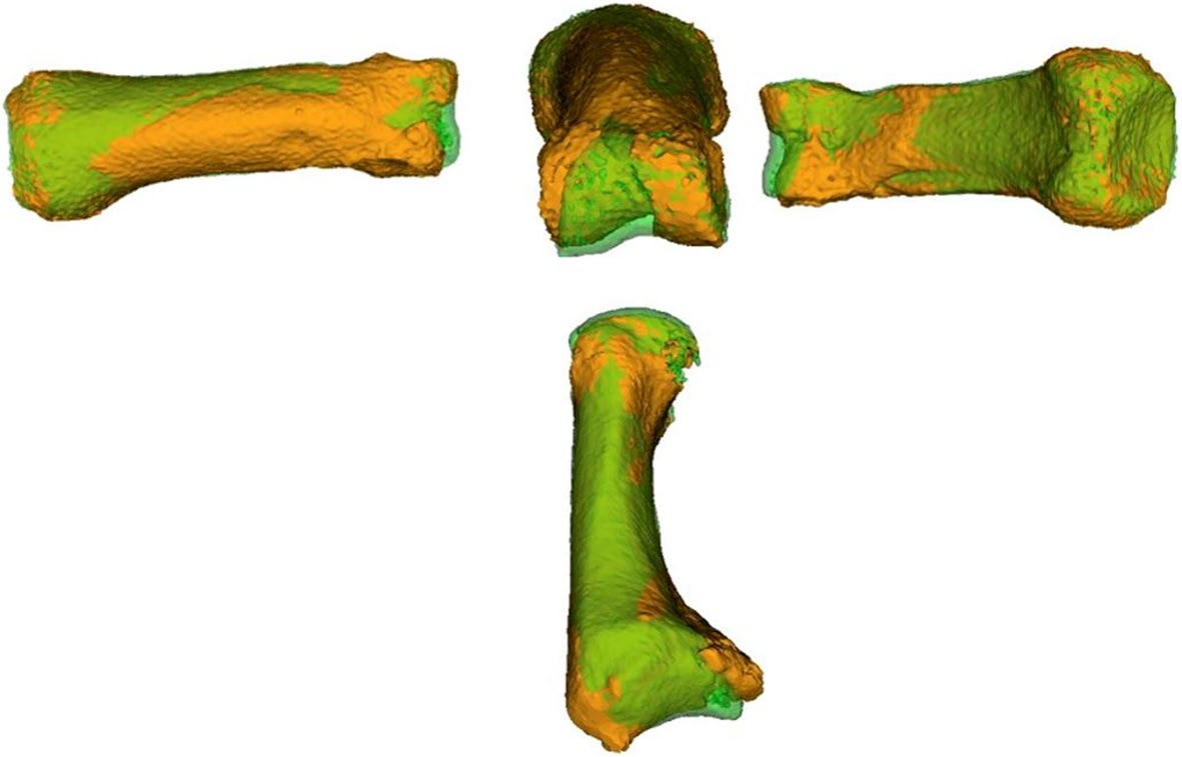
Top: Overlay of the mirrored healthy contralateral bone in a proximal phalanx, orange: affected side, green: contralateral side, distal incongruency is visible; bottom: Overlay of the mirrored healthy contralateral bone in a metacarpal I, orange: affected side, green: contralateral side, proximal incongruency is visible

**Fig. 3:**
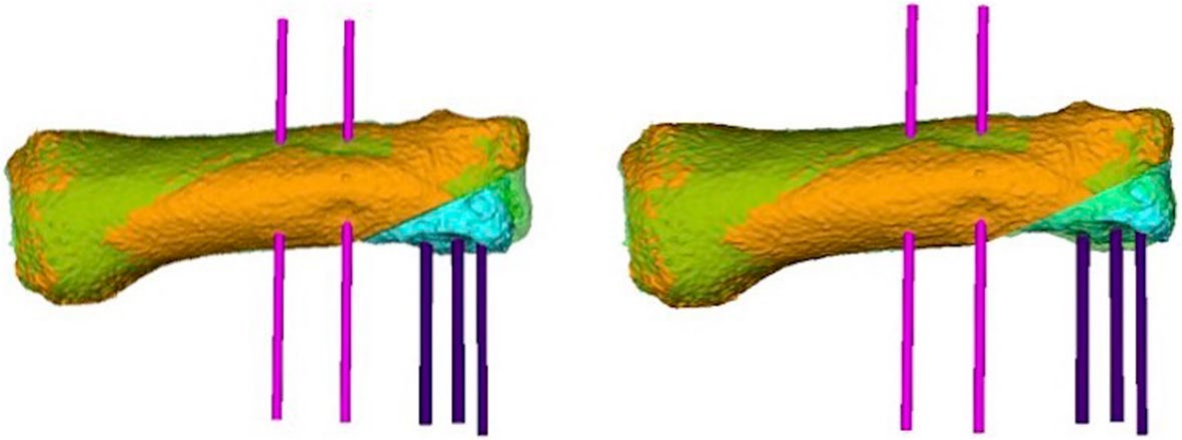
Quantification of malposition and reposition, blue: osteotomy fragment, left: before the osteotomy, right: after the osteotomy

Quantification of the deformity in comparison to the contralateral healthy side and planning of the surgical correction was executed with the in-house developed software CASPA (Balgrist, Zurich, Switzerland), which enables one to use computer-aided design (CAD) functions. For this purpose, initial volumetric fitting, using the overlay of the mirrored contralateral side (green) over the malunited bone (orange) (Fig. 2), was performed followed by quantification of malposition, as previously described [4]. To restore joint congruency, fragment reorientation, including shortening, lengthening or in combination with additional closing- or open wedge were simulated for each case (blue) (Fig. 3). The surgical goal was to restore joint congruency, while preventing extensive surgical dissection or opening of the joint capsule if possible. 3D-planning of joint congruency was achieved by moving the fragment in the three different axis of a coordinate system until intraarticular steps are removed and joint congruency compared to the contralateral side is achieved. This goal has been considered during the meticulous preoperative analysis of the malposition and the surgical CAD planning. Preoperative planning was performed by the executing hand surgeon (A.S.). Depending on the complexity of the case, the required planning time takes about 20–40 minutes. The financial expense per patient was approximately 250 € with 200 € for the 3D-printing of the polyamide parts and about 50 € for the sterilization process. In-house printing of the guides, which are made out of polyamide (nylon), was performed with an EOS© laser sintering printer.

### Surgical technique and postoperative care

**Fig. 4:**
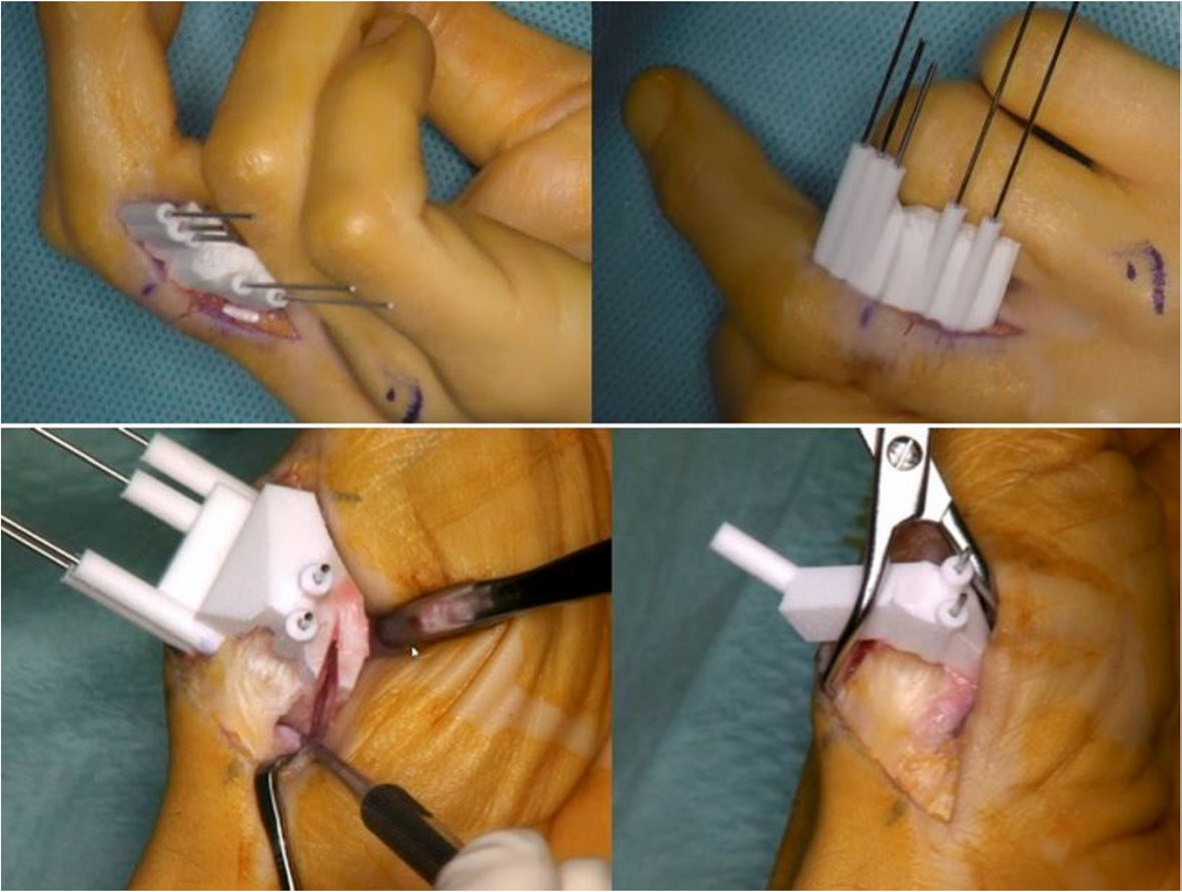
Top: Positioning of bone surface contact guide previous to osteotomy without arthrotomy in a proximal phalanx; bottom: Intraoperative positioning of bone surface contact cutting and reposition guide in a metacarpal I

**Fig. 5:**
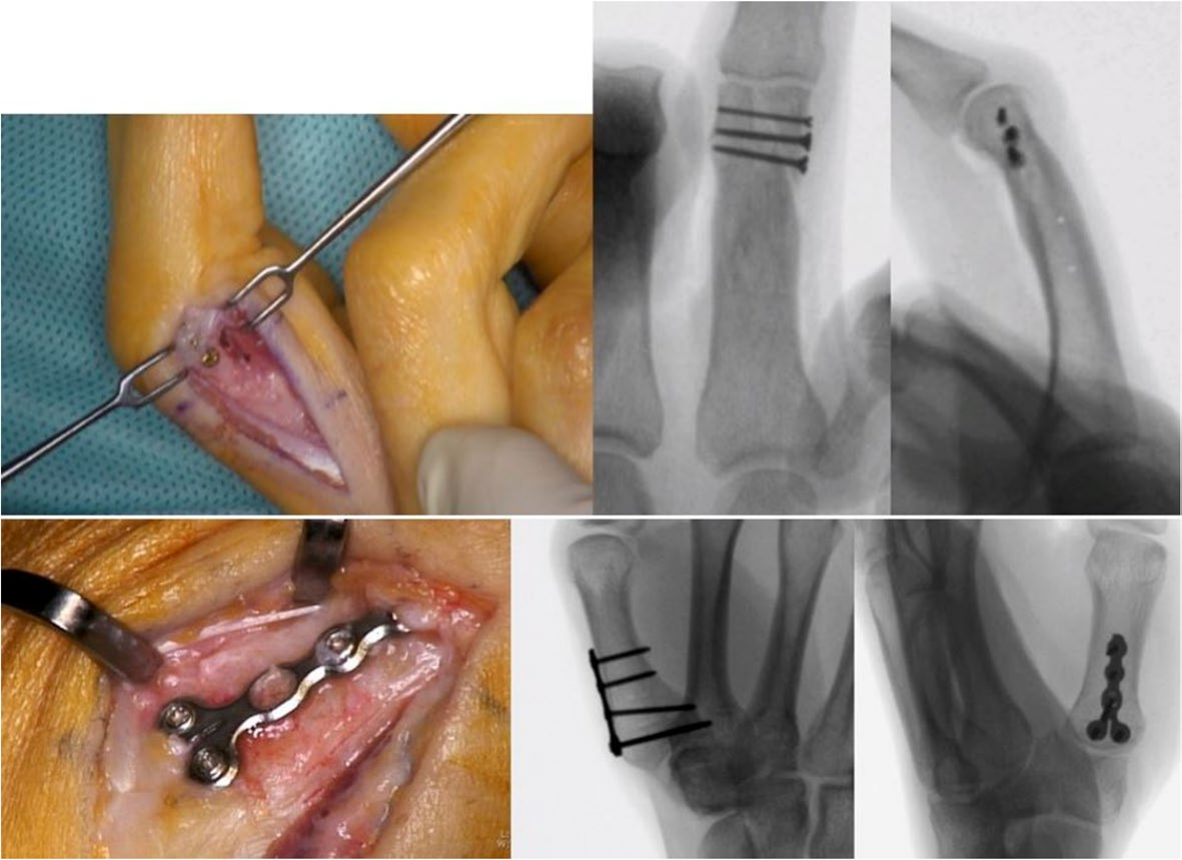
Top: Left: Intraoperative screw fixation of reposition. Right: Image intensified radiographic examination after reduction and fixation; bottom: Left: Intraoperative plate fixation of reposition. Right: Image intensified radiographic examination after reduction and fixation

All corrective osteotomies were performed by one senior hand surgeon (A.S., expert level [10]) in regional or general anesthesia. Depending on the complexity of each case, at least one cutting and one reposition guide was used for every operation. The appropriate surgical approach was chosen depending on the location of the deformity. After detaching the soft tissue off the bone, the bone surface contact sawing guide was positioned and fixated with reference Kirschner (K)-wires (Fig. 4). Full contact to the bone needs to be assured and verified visually. Positioning of the sawing and reposition guides were referenced on 3D printed bone models of each individually prior to the osteotomy. The osteotomy was performed through the defined slits of the sawing guides. After removal of the osteotomy guides and retaining of the reference K-wires, any bony cut-off that may have occurred was removed. The degree of single- or multiplanar correction that partially included wedge osteotomies, was defined preoperatively and achieved with the help of the reposition guide. Those guides were positioned onto the reference K-wires. Autologous cancellous bone grafting (from offcut or distal radius) was added if needed. The reduction was stabilized with an internal screw or plate fixation and controlled visually and with intraoperative x-ray (Fig. 5). Whenever possible, the periosteum was included in the closure over the implanted material and the operation site was closed with respect to the different layers. Aftercare was defined by the patient-specific pathology and usually included immobilization and unloading for four to six weeks postoperatively.

### Radiological and clinical examination

**Fig. 6:**
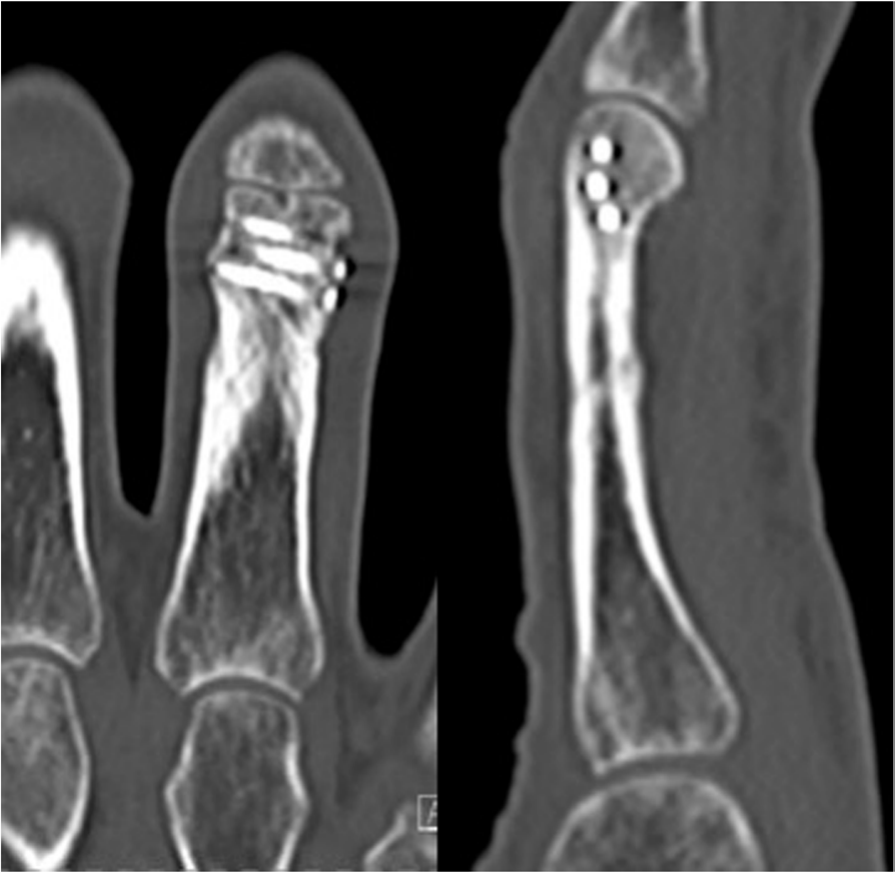
CT scans two months postoperatively showing joint congruency and consolidation

Patients were seen regularly for postoperative follow-up examinations after six to eight weeks, three months and one year postoperatively. Further follow-up examinations took place on patients request of in cases of subjective or objective dissatisfaction. In addition, follow-up CT scans were performed six to eight weeks after surgery (Fig. 6). The intra-articular step-off was measured as the maximum intra-articular step in the sagittal or coronal CT scan as described by Roner et al. [7] by a blinded observer (M.K.) to the functional outcome and patient care. After confirmation of osseous consolidation, mobilization and weight-bearing were initiated.

**Fig. 7:**
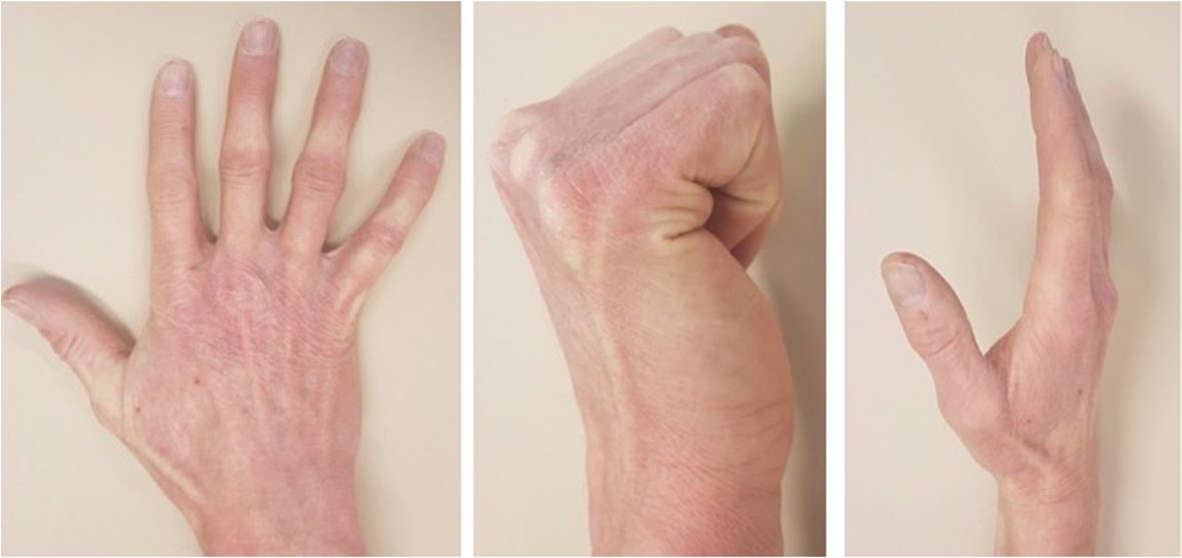
Clinical examination three months after surgical intervention in the proximal phalanx of the third digit

Further follow-up consultations to monitor ROM (Fig. 7), grip strength, and objectifiable deficits took place. The ROM of the affected digit was measured using a goniometer in the neutral-zero-method. Grip strength was assessed using a dynamometer (Jamar, Smith & Nephew, Memphis, USA). Complications and irritation due to hardware was documented and its removal was performed as needed. Follow-up examinations were terminated when patients demonstrated functionality equal to the contralateral side, unrestricted ROM and return to work and sport activities.

### Patient-reported outcome measures (PROM)

Patients were contacted and asked to retrospectively recall the situation prior to the corrective osteotomy to then rate their preoperative and current Single Assessment Numeric Evaluation (SANE) score [11], (range: 0–100). Furthermore, the brief Michigan Hand Questionnaire (MHQ) [12] was collected. One patient could not be contacted despite repeated efforts and thus his PROM results were not included.

### Statistics

Data are presented as mean ± standard deviation (SD) and range. The Wilcoxon signed-rank test was used to analyze the difference between the grip strength of the affected and the unaffected side, the articular step as well as for analysis of the Single Assessment Numeric Evaluation (SANE) score. The significance was set <.05. Data were analyzed with SPSS version 23 (SPSS Inc., Chicago, IL, USA).

## Results

### Baseline characteristics and preoperative planning

**Fig. 8:**
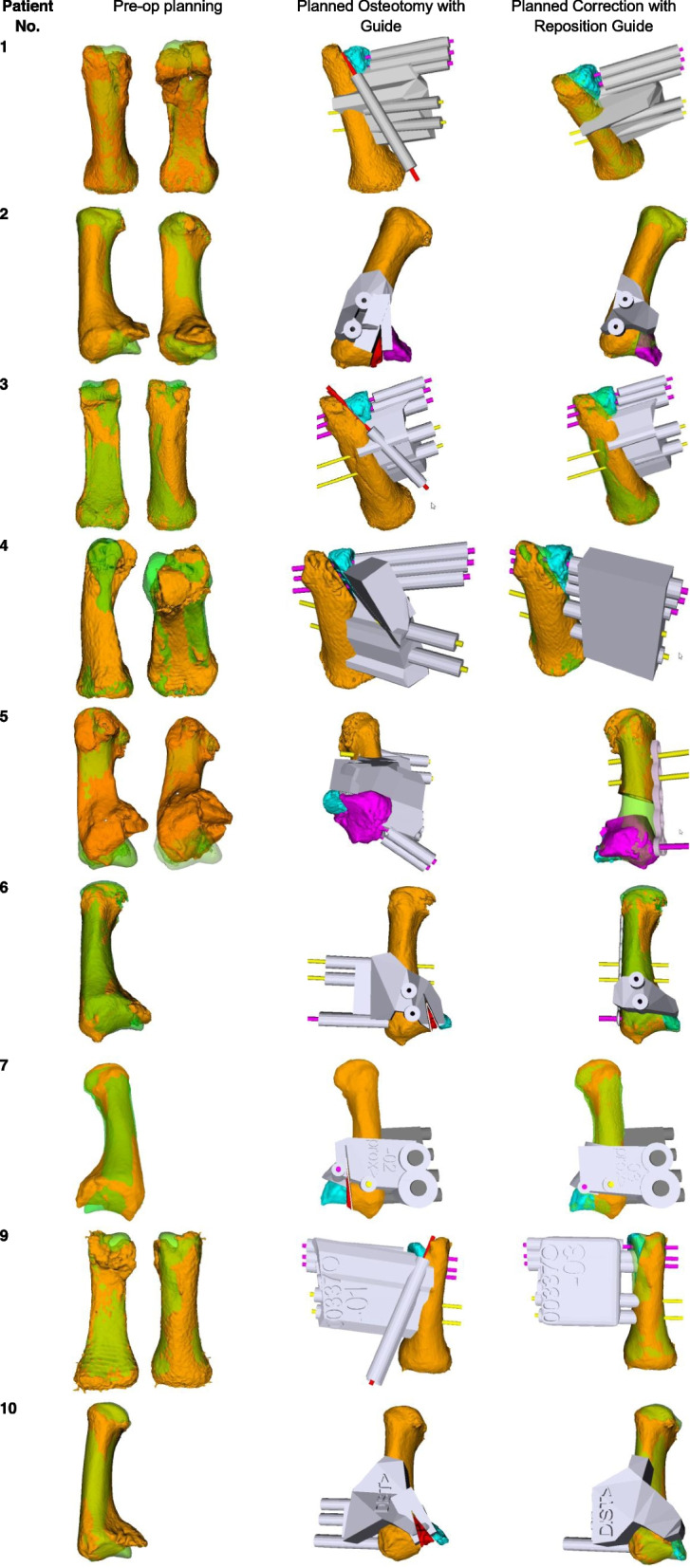
Computer-assisted planning

Ten intra-articular corrective osteotomies were performed at our institution with a mean follow-up of 21 ± 18 (range 3–59) months. The included patients had a mean age of 28.4 ± 12.8 (13.8–51.3) years. Table 1 gives an overview of the demographic and surgical data, including localization and technique of the completed osteotomies. During the preoperative planning of one case, due to a very small fragment size and distal location as well as required tangential correction, the responsible hand surgeon decided to do the correction in a “free-hand technique”.. Due to this, the intra-articular correction was performed using a 3D printed model of the malunited and the corrected bone. Figure 8 shows the results of the computer-assisted planning for nine out of ten patients including the preoperative malformation (orange) and the mirrored healthy area (green), the drill- and/or cutting guide with the malunited fragments (blue) and the reposition guide with the planned fragment position.

**Table 1:**
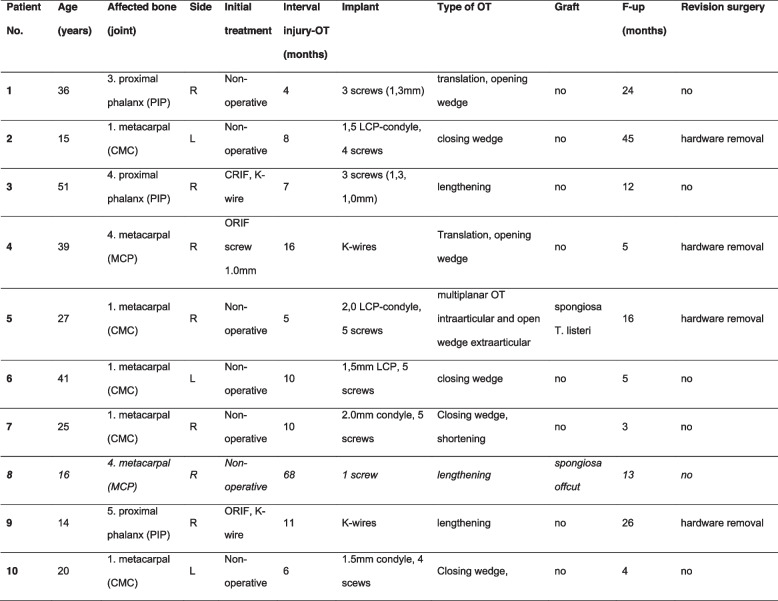
Demographic and surgical data

### Radiological and clinical outcome

All postoperatively performed CT scans, which were completed after an average of 61 ± 8 (54–78) days showed osseous consolidation and joint congruency. The mean articular step was reduced significantly from 1.7 ± 0.6 (0.3–2.4) mm to 0.4 ± 0.3 (0–1) mm (*p* = 0.007). In one patient (patient 9, Table 1) consolidation was observed using only conventional radiographs due to the young age (13.8 years) at time of operation.

Results of the pre- and postoperative ROM and grip strength are listed in Table 2. The mean postoperative grip strength of the operated side (46.7 ± 8.8, 35–60) kg) did not differ from the healthy contralateral side (47.8 ± 8.1, 35–58) kg) (*p* = 0.82) at latest follow-up. All patients with involvement of the PIP joint showed minimal extension deficit of either the distal- or the interphalangeal joint at the latest follow-up examination. None of the patients reported any restrictions during work or leisure time.

**Table 2:** Pre- and postoperative range of motion and grip strength measurements

Patient No.	Preoperative					Postoperative				
	ROM F/E°			Grip power (kg)		ROM F/E°			Grip power (kg)	
	MP/CMC I	PIP / IP	DIP	Affected	Not Affected	MP/CMC I	PIP/ IP	DIP	Affected	Not Affected
1	85/0/0	80/40/0	60/0/0	25	38	100/0/20	80/0/0	45/10/0	35	35
2	NA	NA	NA	NA	NA	0/0/60	20/0/60	NA	NA	NA
3	normal	100/20/0	normal	NA	NA	normal	100/10/0	45/0/0	45	42
4	normal	normal	35/25/0	NA	NA	normal	normal	30/15/0	NA	NA
5	40/0/15	90/0/20	NA	NA	NA	55/0/20	90/0/25	NA	52	58
6	40/0/45	normal	NA	NA	NA	40/0/45	normal	NA	48	52
7	normal	NA	NA	NA	NA	40/0/20	NA	NA	60	50
8	normal	NA	NA	NA	NA	NA	110/0/0	NA	NA	NA
9	100/0/30	45/30/0	45/0/0	NA	NA	90/0/10	60/15/0	90/10/0	NA	NA
10	60/0/0	80/0/30	NA	30	42	70/0/0	NA	NA	40	50

*ROM*: Range of motion in neutral-zero-method, *F/E*: Flexion/ Extension, *MP*: Metacarpophalangeal joint, *CMC I*: Carpometacarpal I joint; *PIP*: Proximal interphalangeal joint, *IP*: Interphalangeal joint, *DIP*: Distal interphalangeal joint, *NA*: Not applicable

No superficial or deep wound infection was documented postoperatively. Due to implant-related irritation, four patients underwent implant removal after a mean time of 15 ± 20 (2–45) months.

### Patient-reported outcome measures

The follow-up interview took place at 72 ± 20 (44–114) months postoperatively. One patient could not be contacted despite repeated efforts. The mean preoperative SANE score was 44 ± 23 (0–70) and increased significantly to 82 ± 12 (60–90) postoperatively (*p* = 0.007). The mean postoperative brief MHQ for the affected hand was 92 ± 8 (71–98) out of 100 points.

## Discussion

With the results presented in this study we could demonstrate the precision of the previously described patient-specific technology [4, 6] to restore joint congruency of intra-articular malunited finger joints. Furthermore, we could confirm the feasibility to perform an intra-articular correction using 3D PSI at the small and delicate structures of the finger joints. By applying this technology, functional and patient-reported.

### Outcome improved in most cases

Intra-articular fractures need to be corrected precisely with respect to the original joint anatomy, which otherwise is associated with pain and an inferior functional outcome [13]. Detailed quantification of the preoperative joint situation and the use of 3D patient-specific guides have shown to be helpful in various situations such as treatment of malunited fractures and complex situations in various joints [5, 6, 14–18]. When involving the articular surface, residual intra-articular deformity is not well tolerated [19, 20] and accurate reconstruction of joint congruency is of upmost importance. Since studies using PSI for intraarticular correction of postoperative malformation are lacking, comparison and discussion of results is not possible. However, as with every patient-specific technology, it is most important to reference the guidance on the patient’s anatomy. However, in small bones such as phalanx this is not as simple. Previous studies have demonstrated the feasibility of executing this technique with excellent precision, without compromising accuracy [4]. A correction of a malunion of 1.4 mm on average was corrected to a malunion of 0.4 mm, which corresponds very closely to the results of our study. Compared to freehand techniques, PSI has the advantage of good reproducibility and accurate osseous quantification of the correction, which has only been done in one previous case with an intra-articular malunion of finger joints.

Surgery at the phalanges are more challenging and prone to complications when compared to the MCs. In MCs it often leads to correction osteotomies, not in the malunited area, but rather in the more proximal, less demanding MC region [21]. This technique can be applied for rotational deformities, not addressing intra-articular problems. It is also challenging to address 3D plane deformity with a single cut osteotomy with no patient-specific guidance, which in this case is required [22]. In addition to intra-articular correction, this technique allows the off-cut to be planned, quantified and removed in a guide-controlled manner. This is particularly important in cases where a Bennett’s fragment is present.

It is interesting to note that extra-articular approaches to intra-articular problems were described early on in order to prevent invasive intra-articular approaches. Similar concepts were described already with external Kirschner-wire fixation of the phalanx, aiming to unload force from the fracture zone of the interphalangeal joints [23]. Although this technique does not open the joint capsule, it has an indirect effect on the intra-articular situation via ligamentotaxis. Besides the technical feasibility, an improved postoperative functionality of the affected fingers could be achieved. The observed deficits in the proximal interphalangeal joint (PIP) extension are within a range that is clinically well tolerated and thus usually do not require further therapy as described previously [24]. In a posttraumatic intra-articular malunited situation, a technique was described by Harness et al. that addresses this situation without capsular penetration [25]. He describes an extra-articular wedge osteotomy with correction of joint alignment and demonstrated an improvement of total digit motion from 154° to 204°. However, it is clear that those techniques do not address the intra-articular malformation, which can then lead to its own complications as described above. Therefore, the authors of this study share the belief the intra-articular situation must be corrected in order to achieve the best result for the patient [26].

Teoh et al. demonstrated in six patients with phalangeal unicondylar malunions a technique, which he corrected with an intra-articular osteotomy [27]. He was able to demonstrate good alignment of the initially deformed joint-line, yet did not quantify the osseous correction and solely used conventional x-rays. His primary goal was to realign the joint-line. He reported an increase in total range of the interphalangeal joint motion from 112° to 155°, yet two of six patients developed a new extension deficit of 10°. Pinal et al. demonstrated the correction of intra-articular malunion via a shot gun approach and extensive reconstruction of the joint in a case series of eleven patients [28]. Despite pain reduction and DASH score improvement, the function worsened in five patients, explained by the authors with the patients’ malcompliance. Yang et al. performed corrective osteotomies of 16 PIP joints using an extensive volar or dorsal approach with following screw- and plate fixation. They reported pain-relief in 14 cases and an increase in ROM from 30.3° to 68.4°, but no PROMs were collected [29]. In a small case series of intra-articular corrective osteotomies for symptomatic Bennett fracture malunions Van Royen et al. were able to decrease the pain from a VAS from 88 to 7 with an increase of grip- and pinch strength [3]. Our results contribute to the previously sparse literature on operative intra-articular correction of malunions. As in previous studies, functionality was improved, and for the first time, osseous correction was precisely quantified. We agree with the authors of many studies that intra-articular malunion must be addressed, but as here presented with the advantages of an extra-articular approach. In our opinion, this novel technique ranks among the current possibilities and advantage of PSI and should be considered when approaching such pathologies.

One limitation of PSI are the associated costs and time needed for surgical planning and production of the surgical guides [4]. In the case of malunions which do not need to be scheduled urgently, we think the benefits of a precise 3D PSI planning and surgical execution outweigh the costs and delay caused by the planning and production of the guides. Furthermore, the present study represents a small case series that needs to be confirmed by broader application and larger cohorts. Short follow-up time does not allow conclusion about osteoarthritis in the long-term. However, all patients reported excellent patient-reported outcome measures after medium-term follow-up of a minimum of three years. In case of present radiologic signs for osteoarthritis it appears that, it has no significant impact on the functional outcome at mid-term follow-up. Another limitation may be the operation by a single surgeon. For better comparability and the uniform intraoperative approach, this may be an advantage for this feasibility study, but needs to be reproducible in a larger scale including more surgeons. Furthermore, the retrospective assessment of the patients function preoperatively represents a certain bias and important limitation concerning the functional outcome. Nevertheless, even though it is not objectifiable retrospectively, all patients reported on clear improvement compared to their situation prior to the corrective osteotomy. Concerning the pre- and postoperative clinical examination results presented in Table 2. has to be mentioned, that certain information are missing limiting the expressive power. However, in our opinion, there is still enough data to be able to give a statement on the improvement the patient have undergone. For the same reason, we decided to include the patient for whom PSI correction of malformation was performed without 3D-planned guides. Technically, it still represents a corrective osteotomy of an intraarticular step. Inclusion of this case might diminish the expressiveness of 3D-planned PSI, but clinically the patient gives us further information on the outcome of an intraarticular correction. This case, due to a very small fragment size which needed correction, may also demonstrate the boundaries of PSI surgery in the phalanges at the current stage. The personal experience showed, that a fragment needs to be at least 4x5x6mm to use PSI guides. Additionally, surface structure is of importance, since edgy surfaces, where fragments can fit into are easier to address. Further scientific investigations are needed to be able to draw conclusions concerning this special problem.

## Conclusion

The authors conclude that by applying this feasible and previously described computer-aided technology intra-articular malposition in the trapeziometacarpal and finger joints can be corrected to achieve improved joint congruency.. An improvement of the functional outcome can be achieved without development of complications.

## Data Availability

The datasets generated and analysed during the current study are not publicly available due to personal information of the included individuals and foreign language but are available from the corresponding author on reasonable request. The authors declare that all data supporting the findings of this study are within the article.

## Abbreviations

3D: Three-dimensional
PSI: Patient-specific instrumentation
CT: Computer-tomography
SANE score: Singe Assessment Numeric Evaluation score
MHQ: Michigan Hand Questionnaire
ROM: Range of motion
MC: Metacarpophalangeal
CMC I: First carpometacarpal joint/trapeziometacarpal joint
mm: Millimeter
CAD: Computer-aided design
K-wire: Kirschner-wire
PROM: Patient-reported outcome measure
SD: Standard deviation
kg: Kilogram
DASH score: Disabilities of the Arm, Shoulder and Hand score
PIP: Proximal interphalangeal joint
ORIF: Open reduction internal fixation
